# Nickel-Based Alloy Dry Milling Process Induced Material Softening Effect

**DOI:** 10.3390/ma13173758

**Published:** 2020-08-25

**Authors:** Jun Zha, Zelong Yuan, Hangcheng Zhang, Yipeng Li, Yaolong Chen

**Affiliations:** 1 School of Mechanical Engineering, Xi’an Jiaotong University, 28 Xianning Road, Xi’an 710049, China; jun_zha@xjtu.edu.cn (J.Z.); chenghangz@foxmail.com (H.Z.); liyipeng@mail.xjtu.edu.cn (Y.L.); chenzwei@mail.xjtu.edu.cn (Y.C.); 2 Shenzhen Research School, Xi’an Jiaotong University, Hi-Tech Zone, Shenzhen 518057, China

**Keywords:** dry milling, nickel-based alloy, material softening effect, ceramic cutting tools

## Abstract

Improving the cutting efficiency is the major factor for improving the processing of nickel-based alloys. The novelty of this research is the calibrated SiAlON ceramic tool dry milling nickel-based alloy process. Firstly, the nickel-based alloy dry milling process was analyzed through the finite element method, and the required milling force and temperature were deduced. Then, several dry milling experiments were conducted with the milling temperature, and the milling force was monitored. The change in cutting speeds was from 400 m/min to 700 m/min. Experimental results verified the reduction of the dry milling force hypothesized by the simulation. The experiment also indicated that with a cut depth of 0.3 mm, cut width of 6 mm, and feed per tooth of 0.03 mm/z, when milling speed exceeded 527.52 m/min, the milling force began to decrease, and the milling temperature exceeded the nickel-based alloy softening temperature. This indicated that easy cutting could be realized under high-speed dry milling conditions. The interpolation curve about average temperature and average milling forces showed similarity to the tensile strength reduction with the rise of temperature.

## 1. Introduction

A nickel-based superalloy refers to an alloy with high strength and good resistance to oxidation at high temperatures together with good creep behavior, as Suárez et al. [1] and Polvorosa et al. [2] stated. Yu et al. [3] and Marques et al. [4] reported that these alloys have been widely used in gas turbines, aero engines, and other key components in the aeronautics industry, and the machining productivity improvement has attracted great research attention [5]. However, the nickel-based alloy machining process is quite arduous, as Thakur et al. [6] stated, which implies high specific cutting force, exceeding the value of 3000 N/mm^2^, as Polvorosa et al. [7] and Suárez et al. [8] researched. Xavior et al. [9] commented that a large amount of cutting heat was generated at the shear region, implying premature tool wear [10]. Ezugwu et al. [11] proposed that under high-temperature conditions, it is easy to weld the processing material onto the tool surface, which not only affects the life of the tool but also has a great influence on the surface quality of the machined substance. Zhang et al. [12] stated that discovering how to obtain better surface quality while improving the cutting efficiency of nickel-based alloys has become an urgent problem to be solved in the field of nickel-based superalloy machining.

Klocke [13] proposed that tool wear has become a major factor limiting the efficiency of superalloy processing. Bhatia et al. [14] suggested that the cutting edge of a tool is prone to wear at high speed under high-temperature conditions. Habeeb et al. [15] studied the machinability of nickel-based alloy 242 by using different tool materials under high-speed cutting conditions and found that the main failure mode of the tool is chipping in the high-temperature shear zone. When turning pure nickel, Tan et al. [16] found that a high cutting temperature is the main factor leading to the failure of cutting tools, which can lead to low efficiency in the milling process.

Cutting nickel-based alloys inevitably lead to higher temperatures than when milling other materials. During the machining process, cutting heat affects a major part of the cutting tool wear. However, nickel-based alloys also have characteristic tensile strength and yield strength, which decrease with increasing temperature. This property is called the thermal softening effect of nickel-based alloys.

Laser-assisted machining (LAM) is based on such a property and can remove more hard-to-cut material than other methods due to its higher cutting efficiency. Liao et al. [17] used carbide tools to cut Inconel 718. Due to the increase of the cutting temperature caused by this method, the precipitation phase γ’ of the nickel-based alloy at high temperature emerges, which decreases the hardness of the superalloy. The softening effect occurs during processing when the temperature is raised to 650 °C. Sun et al. [18] proposed that the cutting shear zone temperature could reach 600–650 °C. Garcí et al. [19] suggested that when such temperatures are used in the cutting process in combination with higher cutting speed and feed rate, the result will be a higher removal rate of the cutting material. López de Lacalle L.N. et al. [20] put forward the plasma-assisted milling for three low machinability alloys, and nickel-based alloys could be processed under high cutting speeds with the improvement of tool life. However, Leopardi et al. [21] found that due to the radiation of the laser and the higher temperature in the cutting area, the tool used for cutting could be affected, causing the tool to wear more easily.

Ceramic tools have good thermal stability, resulting in performing very well during high temperature cutting processes. The widely used heat-resistant high-temperature ceramic materials are mainly silicon nitride, aluminum oxide, boron nitride, and so on. Liu [22] put forward that silicon nitride ceramics are resistant to oxidation and corrosion at high temperatures. Through experiments, it is shown the ceramic tool is made of an α/β-SiAlON material, and the whisker-reinforced ceramic tool has similar performances during the cutting process.

Although ceramic materials have been used in tools when turning nickel-based superalloys for a long period of time, and some milling cutters have already used ceramic inserts in the milling process [23], the dry milling process by monolithic ceramic tools has been less explored. Dry milling is an environmentally friendly method in manufacturing. When using the monolithic ceramic milling tool (SiAlON) made according to the US patent US9481041B2 of Kennametal Inc [24], the base material is mainly Si_3_N_4_. The material removal rate of the nickel-based superalloy Inconel 718 can reach up to 14,500 mm^3^/min, under a cutting speed of 720 m/min, which could lead to a high material removal rate of the nickel-based alloy, further increasing the milling efficiency of this hard-to-cut material.

In this research, high speed and high-efficiency machining method for nickel-based alloy was proposed to solve the problem of low material removal rate caused by cutting force and excessive cutting heat during the dry milling process. Because the next cutting process could be affected by the previous step, the heat generated on the workpiece surface and sub-surface could increase and soften the workpiece. When the workpiece material softened, the workpiece material could be easily cut from the buck material. To prevent the damage to the milling cutter caused by extra heat, a full body ceramic tool was used. First, the dry milling process was modeled by a finite element model (FEM) model and mechanistic model to simulate the milling process. Then, through detecting the heat generated in the actual milling process and comparing the force value in the experiments, a brief understanding of the high-speed ceramic dry milling process was acquired. The high-speed dry milling process of nickel-based alloy showed a thermal softening effect for the workpiece material. By using the thermal softening effect, a high material removal rate of the nickel-based alloy was achieved, which could increase the milling efficiency of nickel-based alloy.

## 2. Force Model and Milling Temperature Simulation

The material used in the model was Inconel 718, and the milling cutter proposed was 12 mm in diameter with six flutes made of a SiAlON ceramic.

### 2.1. Mechanistic Milling Force Model

Owing to the spiral shape of the milling tool, the cutting process is usually accompanied by periodic variation of the cutting force. The magnitude of cutting force is determined by tool material, cutting material, cutting parameters, and so on. According to the cutting theory presented by Altintas [25], the milling force can be modeled. Fernández-Abia et al. [26] also developed a new model to estimate the cutting force in the cutting process. This research has also made a mechanistic model, as the following equation shows.

(1) $$\left\{\begin{array}{l} F_{x}=-\frac{N}{4}K_{tc}ac-\frac{N}{\pi }K_{re}a\\ F_{y}=\frac{N}{4}K_{tc}ac+\frac{N}{\pi }K_{te}a\\ F_{z}=\frac{N}{4}K_{fc}ac+\frac{N}{2}K_{re}a \end{array}\right.$$

where the *F_x_*, *F_y_*, *F_z_* in *N* represents the milling force in *x* (feed direction), *y* (radial direction), and *z* (tangential direction), respectively. *a* in mm refers to the cutting depth, and *c* in mm/min refers to the feed rate. *N* refers to the number of flutes. The *K_tc_*, *K_rc_*, *K_fc_* refer to cutting force coefficients, which are contributed, respectively, by shearing action in tangential, radial, and axial directions. The *K_te_*, *K_re_*, *K_fe_* refer to edge constants in the same directions as above, respectively.

The cutting force coefficients could be obtained by the mechanistic identification of cutting constants’ experiments, which could be evaluated as Equation (2).

(2) $$\begin{array}{c} K_{tc}=\frac{4{\bar{F}}_{yc}}{Na},K_{te}=\frac{\pi {\bar{F}}_{ye}}{Na}\\ K_{rc}=\frac{-4{\bar{F}}_{xc}}{Na},K_{re}=\frac{-\pi {\bar{F}}_{xe}}{Na}\\ K_{fc}=\frac{\pi {\bar{F}}_{ze}}{Na},K_{fe}=\frac{2{\bar{F}}_{ze}}{Na} \end{array}$$

Since the average cutting force can be obtained through linear regression of the data, the following Equation (3) could be acquired.

(3) $${\bar{F}}_{q}={\bar{F}}_{qc}c+{\bar{F}}_{qe}\;(q\;=\;x,\;y,\;z)$$

${\bar{F}}_{q}$ represents the average cutting force. ${\bar{F}}_{qe}$ refers to cutting force coefficients in *x*, *y*, *z* directions, while ${\bar{F}}_{qe}$ refers to edge force, which does not depend on the chip thickness.

Because of the milling feed rate, *c* in Equation (3) could be calculated, as shown in the following Equation (4).

*c* = *f_z_* × *N* × *S* (4)

In Equation (4), *f_z_* in mm/z refers to feed per tooth, *N* refers to the number of flutes, *S* in mm/min refers to the spindle speed.

Combining the Equations (2) and (4), Equation (5) could be acquired.

(5) $${\bar{F}}_{q}={\bar{F}}_{qc}\times (f_{z}\times N\times S)+{\bar{F}}_{qe}\;(q\;=\;x,\;y,\;z)$$

The cutting speed *V_c_* in m/min could be calculated as Equation (6).

(6) $$V_{C}=\frac{\pi DS}{1000}$$

Combining Equations (6) and (5), the milling force could be represented as Equation (7).

(7) $${\bar{F}}_{q}={\bar{F}}_{qc}\times (f_{z}\times N\times \frac{1000V_{C}}{\pi D})+{\bar{F}}_{qe}\;(q\;=\;x,\;y,\;z)$$

The identification of cutting constants’ experiments was conducted. By detecting the milling force in the *x*, *y*, and *z* directions, a linear equation of milling force could be established. The cutting forces in three directions could be acquired by changing the feed rate in the experiment. Moreover, the cutting coefficients could also be acquired by solving this equation.

**Figure 1:**
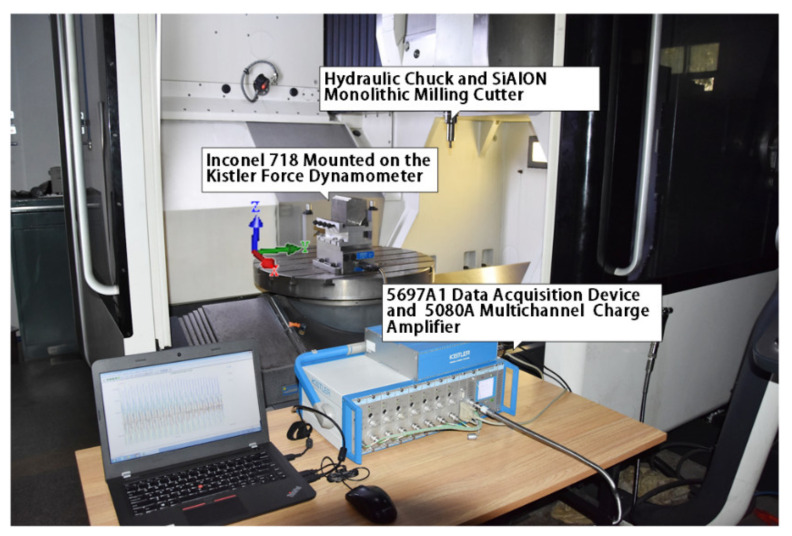
Cutting coefficients identification experiment arrangements.

In the cutting coefficients identification experiments, the 12 mm diameter monolithic ceramic end milling tools and Inconel 718 were put into use to identify the cutting constant. It was conducted on a DMG HSC75 linear high-speed machining center (DMG, Maho, Germany). The cutting force was acquired by a KISTLER force dynamometer 9129AA (Kistler Group, Winterthur, Switzerland). The experiment arrangement is shown in Figure 1.

The spindle speed was set to be 16,000 rpm, cutting parameters were set to change from the feed per tooth 0.015 mm to 0.06 mm, as seen in Table 1, and with other parameters set to be constant. The spindle speed was 16,000 rpm, cutting depth was 0.45 mm, and the cutting width was 6 mm in the cutting coefficient identification experiments.

Through liner regression of the parameters and force data, milling parameters ${\bar{F}}_{qc}$ and ${\bar{F}}_{qe}$ (*q* = *x, y, z*) could be acquired by solving the binary linear equation in Equation (7) to get ${\bar{F}}_{qc}$ and ${\bar{F}}_{qe}$ (*q* = *x, y, z*) and then brought them into Equation (2) to get *K_tc_*, *K_rc_*, *K_fc_* and *K_te_*, *K_re_*, *K_fe_*. The results are listed in Table 2. Because the edge constants were the result of the cutting constant in the equation, these constants had units of 1. The native sign of *K_tc_* and *K_te_* in Table 2 represents the reversed force direction.

**Figure 2:**
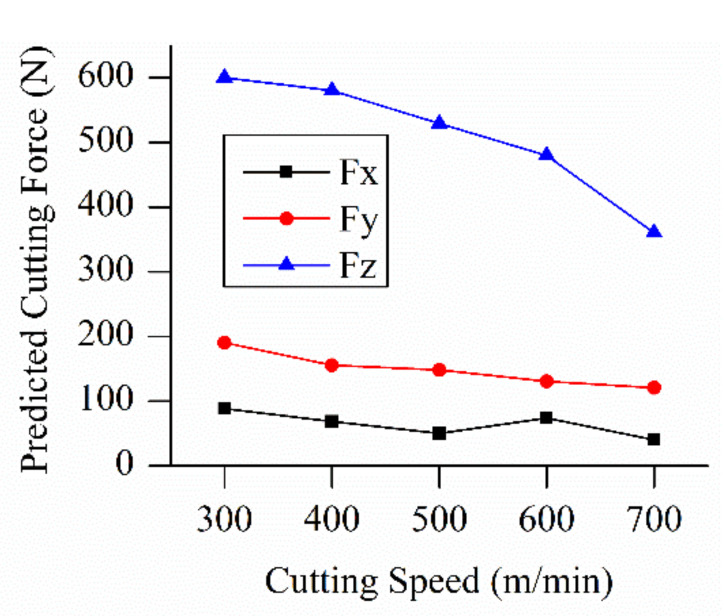
Predicted force values accompanied by the rise of cutting speed.

Through the identification experiments, the mechanic milling force model was established. Setting the milling depth of cut as 0.3 mm, the number of flutes as 4, the cutting width as 6 mm, and the feed per tooth as 0.03 mm/z, with varying milling speed (300 m/min to 700 m/min) by changing the spindle speed value, the predicted force value results are listed in Figure 2. The tangential, radial, and axial force all caused a reduction of milling force with the rise in cutting speed. The cutting force in *x*, *y,* and *z* directions all caused a reduction when cutting speed rose.

### 2.2. Dry Milling Temperature Simulation

Milling temperature is always difficult to predict and detect during experiments. In this research, the AdvantEdge (Third Wave Systems, Minneapolis, MN, USA) software was used to simulate the milling temperature in the milling shear region. The parameters of milling cutter and simulation parameters are listed in Table 3.

In order to calculate the dynamic flow stress, He et al. [27] proposed using the J–C (Johnson–Cook) constitutive model. It was proposed by Johnson and Cook (1983) and is shown in Equation (8).

(8) $$\sigma =(A+B\varepsilon^{n})\left[1+C\ln(\frac{\dot{\varepsilon }}{{\dot{\varepsilon }}_{0}})\right]\left[1-{\left(\frac{T-T_{r}}{T_{m}-T_{r}}\right)}^{m}\right]$$

In Equation (8), *σ* is equivalent stress, *A* is the yield stress under reference deformation conditions (MPa), *B* is the strain hardening constant (MPa), *ε* is the equivalent plastic strain, *C* is the strain rate strengthening coefficient, *n* is the strain hardening coefficient, and *m* is the thermal softening coefficient. $\dot{\varepsilon }$ is the strain rate, ${\dot{\varepsilon }}_{0}$ is the reference strain rate, *T* is the current absolute temperature, *T_m_* is the melting temperature, and *T_r_* is the reference room temperature (20 °C).

The simulation process was based on a shear plane model, which was a steady-state chip formation process, and the workpiece material was based on a plastic material model.

When the material was cut off from the workpiece, it was necessary to evaluate the process of removal of the material. In the simulation process, a shear failure model was used. In this model, the fracturing of the material was evaluated by equivalent plastic strain changes, as shown in Equation (9).

(9) $${\bar{\varepsilon }}_{f}{}^{pl}=\left[d_{1}+d_{2}\;\exp\left(d_{3}\frac{p}{q}\right)\right]\left[1+d_{4}\ln\left(\frac{{\dot{\bar{\varepsilon }}}^{pl}}{\dot{\varepsilon }}\right)\right]\left(1+d_{5}T^{*}\right)$$

${\bar{\varepsilon }}_{f}{}^{pl}$ is the equivalent failure plastic strain, *p* is the compressive stress, *q* is the Mises stress, *d_1_~d_5_* are failure parameters, ${\dot{\bar{\varepsilon }}}^{pl}$ is the equivalent plastic strain, and T* is the reference temperature.

The fracture value *ω* was defined as.

(10) $$\omega =\sum \left(\frac{\Delta {\bar{\varepsilon }}^{pl}}{{\bar{\varepsilon }}_{f}{}^{p}}\right)$$

where $\Delta {\bar{\varepsilon }}^{pl}$ is the equivalent plastic strain of increment step, and ${\bar{\varepsilon }}_{f}{}^{p}$ is the strain failure value. When *ω* ≥ 1, it could be regarded as the cutting material failing. When all the integration points failed, these elements would be deleted from the buck material, and the material was cut off from the workpiece.

The failure parameters, as concluded by Johnson [28], are listed in Table 4.

The Inconel 718 J-C parameters, as concluded by Chen et al. [29], are listed in Table 5.

The mechanical properties of Inconel 718 are listed in Table 6.

During the cutting process, two different friction zones would form in the cutting zone, which was divided into a sliding area and a cohesive area. When the friction force was less than the limit shear force, chips would squeeze the tool, and the cutting temperature rose. Because the heat dissipation rate was low, the workpiece material would cohere onto the contact surface of the tool. When the friction force was greater than the limit shear force, the contact surface would slide. It was easy to dissipate heat in this region because the contact surface was far from the cutting tool. The friction stress could be calculated as Equation (11).

(11) $$\left\{\begin{array}{l} \tau =\mu \sigma_{n}if\;\tau \leq \tau^{*}\;(sliding\;area)\\ \tau =\tau^{*}if\;\tau \geq \tau^{*}\;(cohesive\;area)\; \end{array}\right.$$

where *τ* is the friction stress, *μ* is the friction coefficient, *σ_n_* is the normal stress, and τ* is the maximum shearing stress.

**Figure 3:**
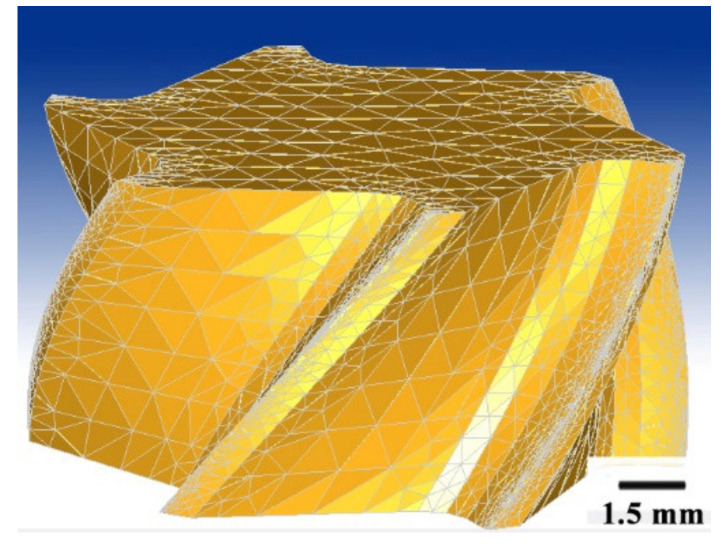
The mesh of the milling cutter.

**Figure 4:**
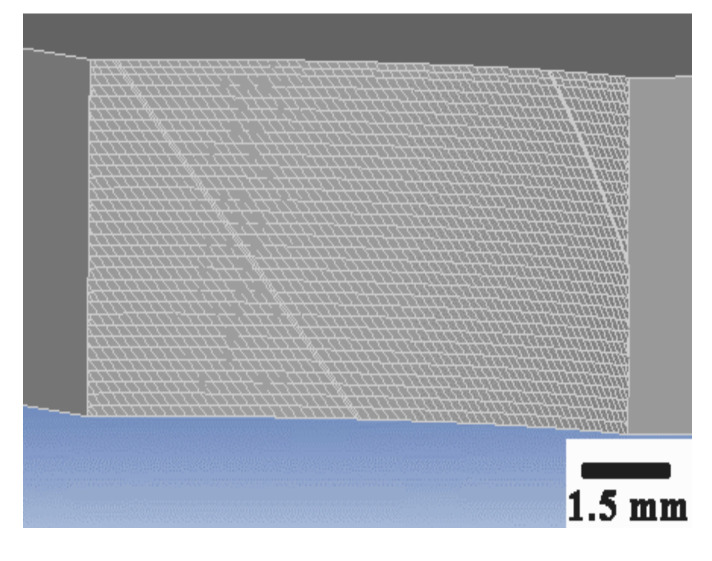
The mesh of workpiece.

The 3-D simulation process minimum size of the element was 0.0117 mm at the chip bulk material, and the minimum element size at the cutter edge was 0.009 mm. Figure 3 and Figure 4 are the mesh of milling cutter and workpiece material.

According to the simulation results, the shear zone thermal map of milling materials and the thermal map of the milling tool could both be acquired.

**Figure 5:**
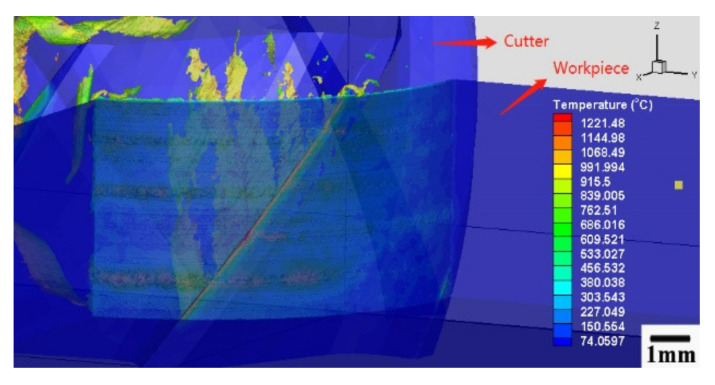
Milling process thermal map of cutting speed 450 m/min, feed per tooth 0.03 mm/tooth, cutting depth 0.3 mm, and cutting width 4 mm.

**Figure 6:**
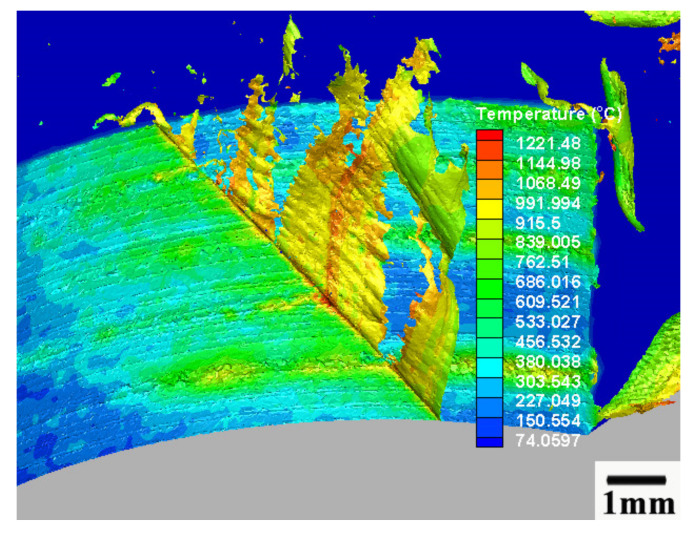
Workpiece shear zone thermal map of cutting speed 450 m/min, feed per tooth 0.03 mm/tooth, cutting depth 0.3 mm, and cutting width 4 mm.

Figure 5 and Figure 6 show the thermal map of cutting speed 450 m/min. Figure 5 presents the temperature distribution on the cutting tool and cutting material, and Figure 6 presents the temperature on the workpiece material. The highest temperature on the milling cutter and workpiece material was 1221 °C. The average temperature on the shear zone was above 900 °C, which was higher than the material softening point of the nickel-based alloy Inconel 718.

**Figure 7:**
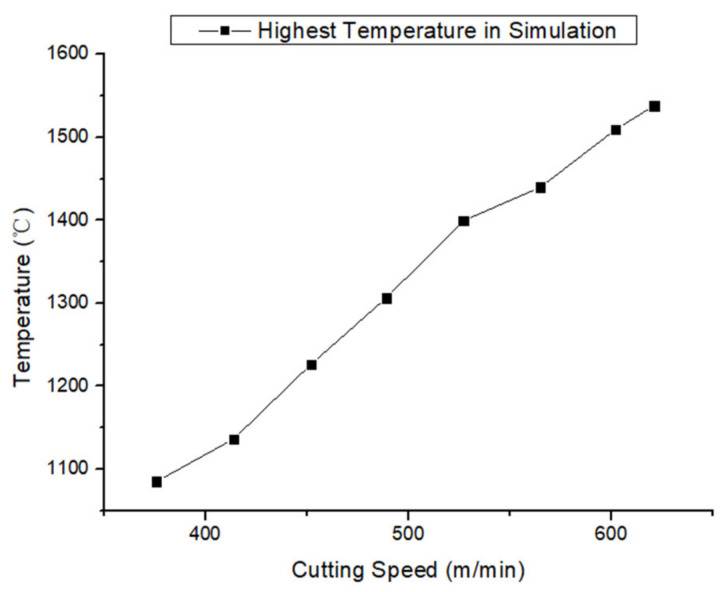
The highest temperature in the simulation process.

The simulation results of the milling temperature in the shear zone with varying cutting speeds are shown in Figure 7. In the simulation process, as the cutting speed rose, the highest temperature at the shear zone increased continually.

According to the milling force and temperature simulation results above (Figure 2, Figure 3, Figure 4, Figure 5, Figure 6 and Figure 7), it can be concluded that with the increase in cutting speed, the cutting force continued to decrease, and at the same time, the cutting temperature rose. The maximum temperature reached 1000 °C above. Thus, as the cutting temperature increased, the material softening effect led to a reduction of cutting force, especially evident in *Fz* direction.

## 3. Experiment Verification

### 3.1. Experiment Design and Set-Up

In the experiments, a DMG HSC 75 linear high-speed machine was used to acquire the higher cutting speed. In order to reduce the random error occurring in the experiments, all the experiments were done twice. The milling cutter’s diameter was 12 mm, with a six-flute monolithic ceramic milling tool. SiAlON was the material used for the milling tool, and its base material was Si_3_N_4_. The tool was manufactured by Kennametal Inc.

**Figure 8:**
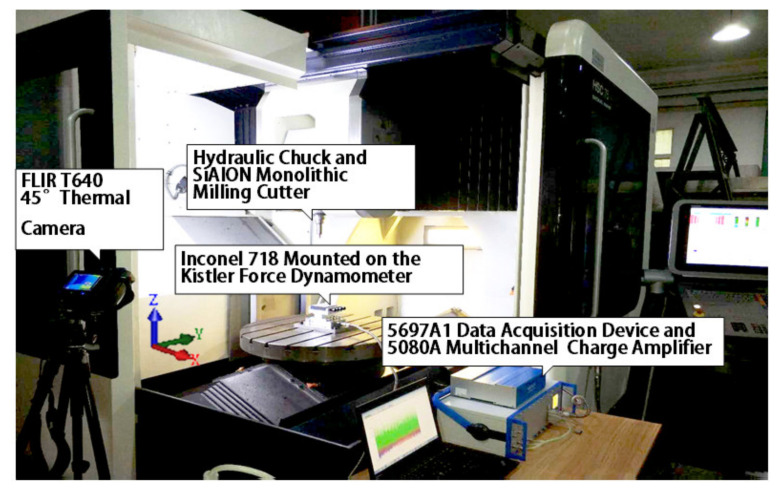
Experimental set-up.

**Figure 9:**
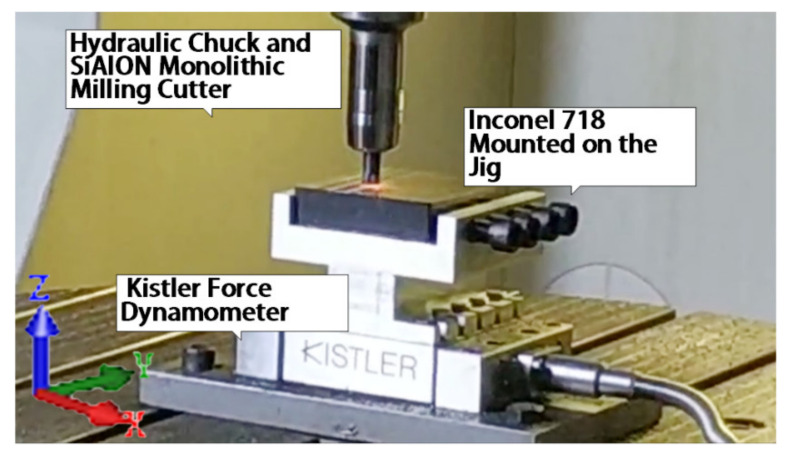
Image of the dry milling process.

As for the experiment parameters, the experiments only changed the spindle speed, which was proportional to the cutting speed. As the cutting speed was in direct proportion to cutting temperature at the shear zone, the change of cutting speed could lead to a change of milling temperature in the shear zone. In this experiment, milling speed was set to varying from 370 m/min to 621 m/min. (Because the highest spindle speed of the machining center was 18,000 rpm, for safety reason, spindle speed was limited to 16,500 rpm. For the 12 mm ceramic tool, cutting speed was limited to 621 m/min). The cutting depth was set to be 0.3 mm, and the cutting width was set to be 5 mm. Because the parameters used in the experiment frequency was lower than the cutting process natural frequency, there was no resonance during the experiment process. Thermal maps were captured by a FLIR T640 45° infrared camera (FLIR, Portland, Oregon, USA). Thermal maps were captured by a FLIR T640 45° infrared camera. The infrared camera could capture thermal temperature ranges from +300 °C to +2000 °C. The infrared camera was mounted on a tripod. The milling force was detected through a Kistler 9129 AA dynamometer (Kistler Group, Winterthur, Switzerland) mounted on a self-designed jig. The experiment setup is shown in Figure 8. The dry milling process is shown in Figure 9 (because the workpiece material was a forging blank, shown as black on its side surface and before the milling experiment, the forging blank on the up and down surface was cleaned to avoid measurement error in the experiments).

### 3.2. Experiment Results and Discussion

The cutting force was obtained from the experiments, and the average cutting force was calculated by Equation (12).

(12) $$F_{ave}=\sqrt{F_{x}^{2}+F_{y}^{2}+F_{z}^{2}}$$

**Figure 10:**
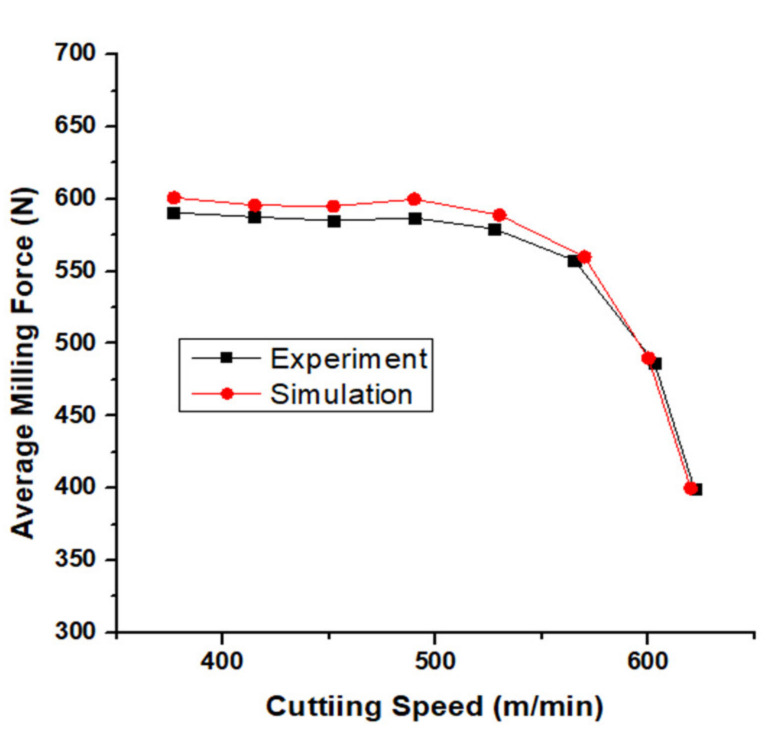
Actual force and simulation force.

As Figure 10 shows, with the rise of spindle speed, the milling force first remained constant until milling speed reached 527.52 m/min (Spindle speed 14,000 rpm), which was a turning point for milling force, after when the force decreased sharply. The behavior of the simulated milling force and the real milling force acquired from the force dynamometer was the same.

Because the experiments were conducted by changing the spindle speeds for the purpose of changing the cutting speed, the experiments could not get an integer number for the cutting speed. The behavior of the milling force in both experiments and simulation was compared. The deviation between the actual milling force and random milling force was 5.37%.

**Figure 11:**
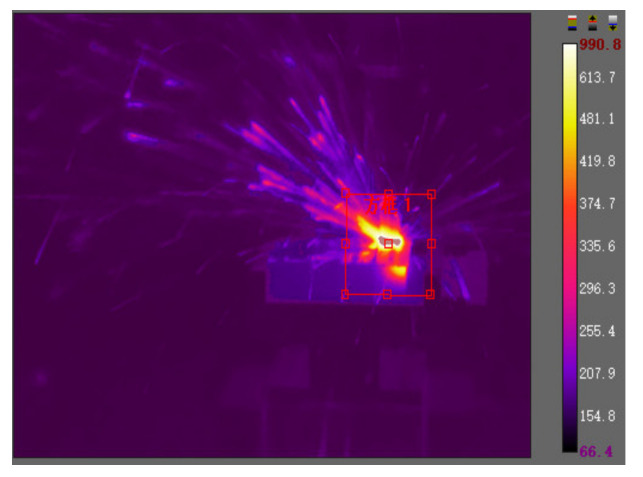
The captured temperature by the thermal camera (cutting speed was 350 m/min).

The captured milling temperature was analyzed by focusing on the square area of the shear zone, as shown in Figure 11. The square area in the thermal map was roughly focusing on the shear zone of the dry milling process. The average temperature in the square area was captured and then analyzed by software, from which a brief understanding of the dry milling temperature in the experiments could be obtained. The FLIR T640 45° thermal camera had an accuracy of ±2 °C, and the accuracy was greatly guaranteed by the thermal camera.

The values measured by the infrared camera were based on the Stefan–Boltzmann law proposed by Cercignani [30], which indicates that the total radiant heat power emitted from a surface is proportional to the fourth power of its absolute temperature.

Equation (13) is the formula of the Stefan–Boltzmann law. In this equation, *T* represents absolute temperature, and *ε* refers to the emissivity. Because the milling temperature was captured through a small square in the cutting region, the emissivity could be considered as a constant. For Inconel 718, when the temperature was 20 °C, Inconel 718′s *ε* was taken to be 0.25. This was set in the thermal camera post-processing software. The software could adjust it automatically when the temperature was changing. *σ_B_* is the Stefan–Boltzmann constant, *σ_B_* = 5.67 × 10^−8^ W/(m^2^·K^4^).

*E = εσ_B_T^4^* (13)

**Figure 12:**
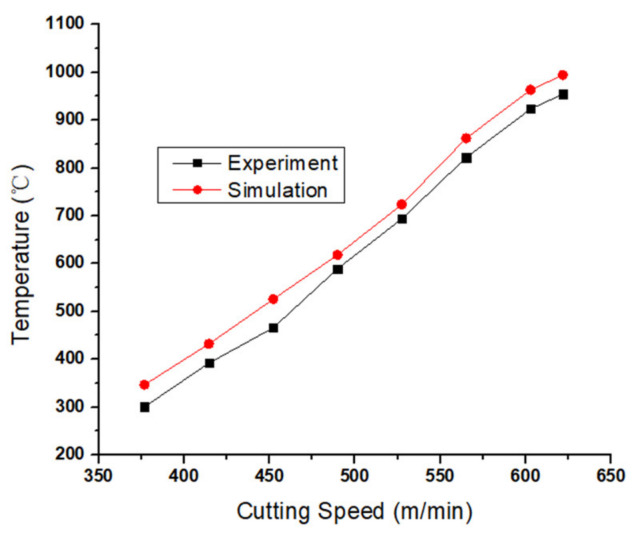
Comparison of average temperature between simulation and experiment.

**Figure 13:**
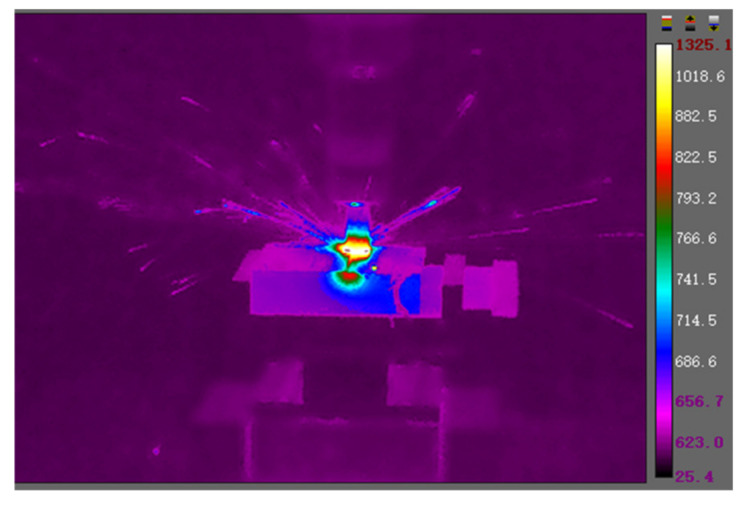
Highest cutting speed thermal map.

In this research, the infrared-camera-captured initial temperature was based on the *ε* value of 1. This primitive temperature was converted by changing *ε* to 0.25. Thus, the actual average temperature value was obtained, and the comparison between the actual value and simulation value with the change of cutting speed is shown in Figure 12, and the thermal image of the highest cutting speed is shown in Figure 13. It is obvious that the average temperature in the simulation was higher than the average temperature in the experiments. The major reason for this phenomenon is that the temperature acquired in the real milling process could not reflect the shear zone’s highest temperature, and the heat dissipation in the environment could lead to temperature value lower than the simulation temperature.

According to the experiments, the tendency of average temperature was to steadily increase with the increase of cutting speed, until when the spindle speed reached 14,000 rpm (cutting speed 527.52 m/min), and the average temperature of cutting region was 653.8 °C. Then, with the improvement of spindle speed, the average temperature in the cutting region exceeded 800 °C, which already exceeded the Inconel alloy material softening point. As shown in Figure 10, when spindle speed reached 14,000 rpm (cutting speed is 527.52 m/min), the cutting force began to decrease and continued to decrease with increasing cutting speed.

**Figure 14:**
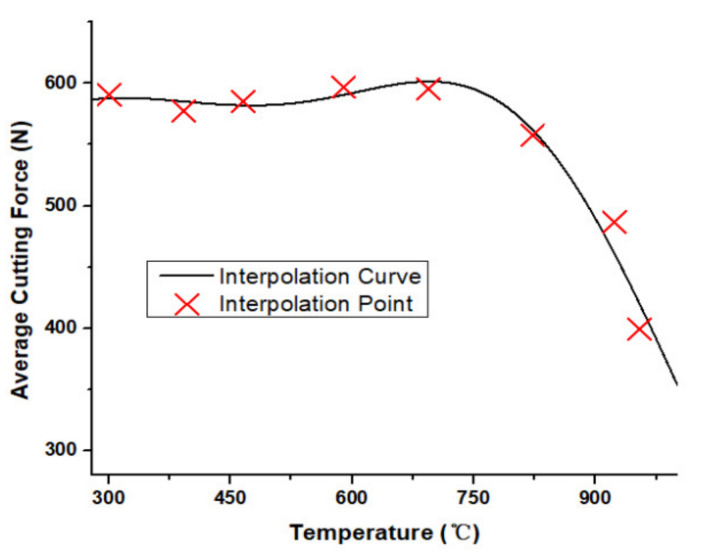
Gaussian interpolation curve.

Given the close relationship between the cutting temperature in the shear zone and the cutting force generated in the machining process, by taking the cutting temperature as the horizontal axis value and the cutting force as the vertical axis value, the scatter plot was drawn, as shown in Figure 14. The two-term Gaussian curve could interpolate the observations. The interpolation curves of Gaussian, the cutting force, and the cutting temperature were obtained. The two-term Gaussian curve was the most precise interpolation curve for the force and temperature. It could clearly reflect the relationship between these two values. The confidence bounds were 95%.

The curve could be expressed by Equation (14). Wherein, t represents the average temperature value.

(14) $$f(t)=341.7\times e^{-{\left(\frac{t-830.7}{295.6}\right)}^{2}+576.9\times e^{-{\left(\frac{t-260.8}{572.7}\right)}^{2}}}\;(N)$$

According to the curve, it can be concluded that when the average milling temperature was at 800 °C, the average cutting force reached a turning point. It indicated when the cutting speed was at 560 m/min, there was a material softening phenomenon on the Inconel 718, which led to the cutting force reduction.

The initial increase of the milling speed induced the average temperature to increase, and the interface between tool and workpiece material might melt and lead to the force reduction. As the temperature continued to increase, more material began to melt and tended to cut more materials, which made the milling depth to increase, as well as increase the milling force. Although the force value fluctuated before the material softening effect, the average milling force at the initial stage almost kept a constant value.

As Figure 14 shows, before the material softening effect happened, the milling force had a relatively constant value. Through calculating the average force value and considering the material softening effect rate, the average milling force before the material softening effect was shown to be 589 N. The force reduction rate (*r*) was calculated as Equation (15), which is *f* (*t*) minus the average force and then divided by the average force. Because it was a division of two force values, so its unit was 1.

(15) $$r=\frac{\left[f(t)-589\right]}{f(t)}$$

**Figure 15:**
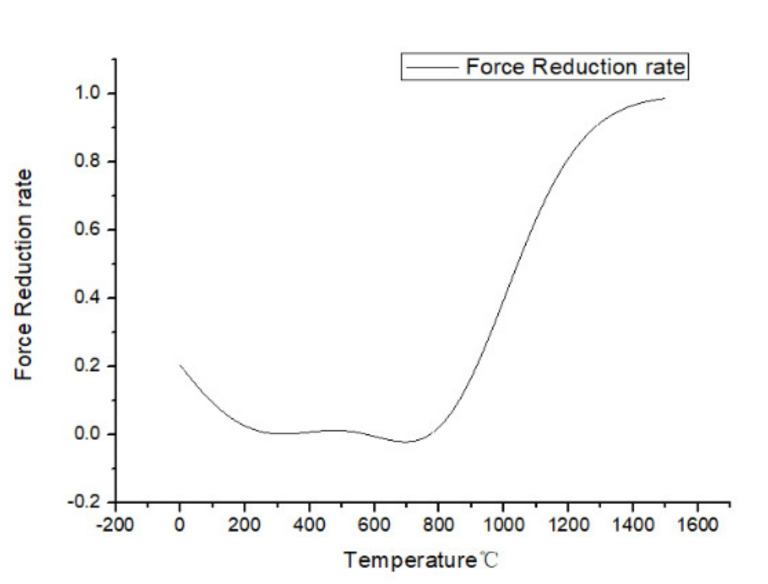
Force reduction rate of Inconel 718 during milling experiments.

It can be concluded in Figure 15 that when the temperature was at 200 °C to 800 °C, the cutting material experienced a work hardening effect. The force reduction started at 800 °C and continuously increased with the temperature thereafter. It can be concluded that when the cutting speed reached a threshold, the cutting heat generated at the machining region was large enough to cause the material softening effect for the nickel-based superalloy.

### 3.3. Tool Wear, Microstructure of Machined Surface, and the Form of Chips Under Different Cutting Parameters

**Figure 16:**
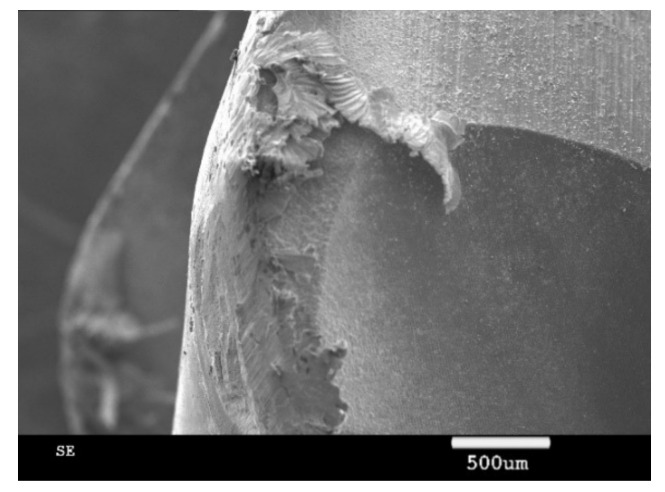
Material bonding on the tool.

**Figure 17:**
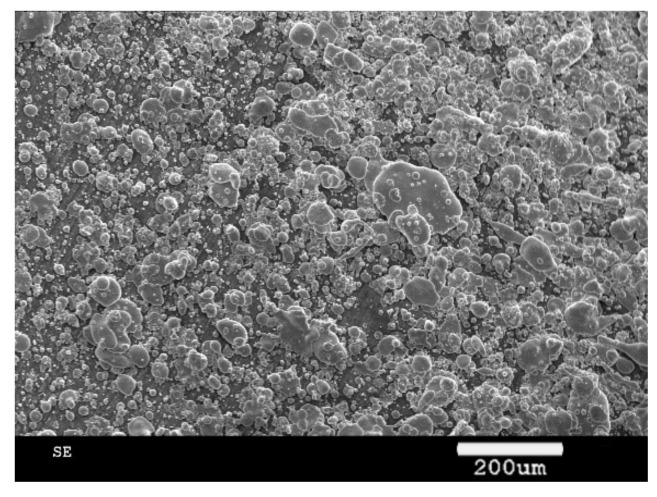
Diffusion wear on the tool surface.

**Figure 18:**
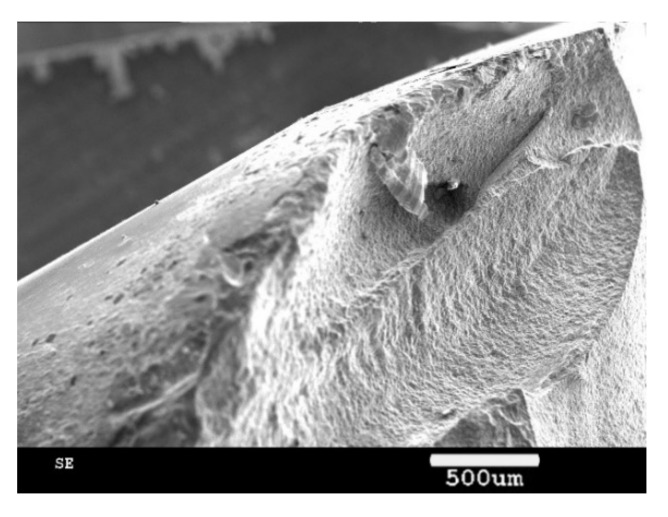
Tipping on the tool’s cutting edge.

The used ceramic tool wear was observed through an SEM (scanning electron microscope, Zeiss, Aallen, Germany), as shown in Figure 16 and Figure 17. The main wears on the ceramic tool when the cutting speed was above 600 m/min were the material bonding and diffusion wear on the tool surface. When the cutting speed was below 600 m/min, especially when the cutting speed was at cutting force turning point, the main tool wear was tipping, as shown in Figure 18.

**Figure 19:**
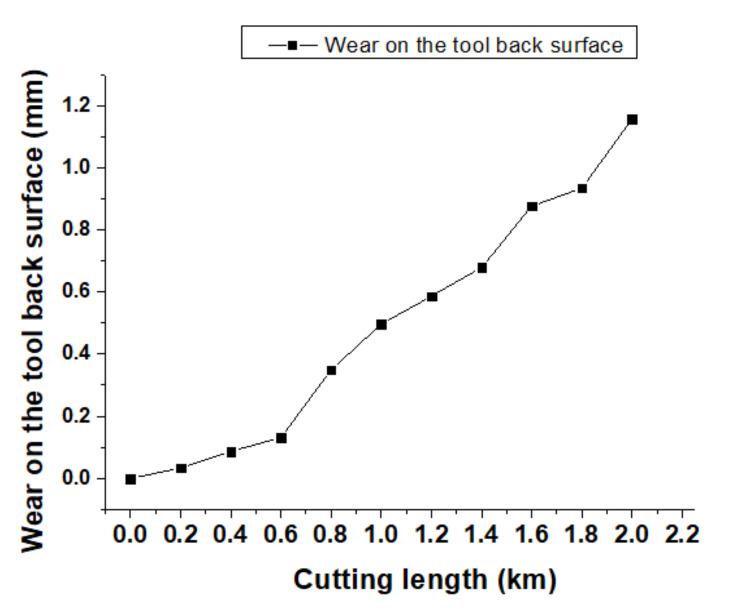
Different cutting lengths induced wear on the tool’s back surface.

When considering the cutting length-induced tool wear, a tool life graph could be drawn, as shown in Figure 19. When cutting length reached 0.6 km, the tool back surface wear began to wear severely.

**Figure 20:**
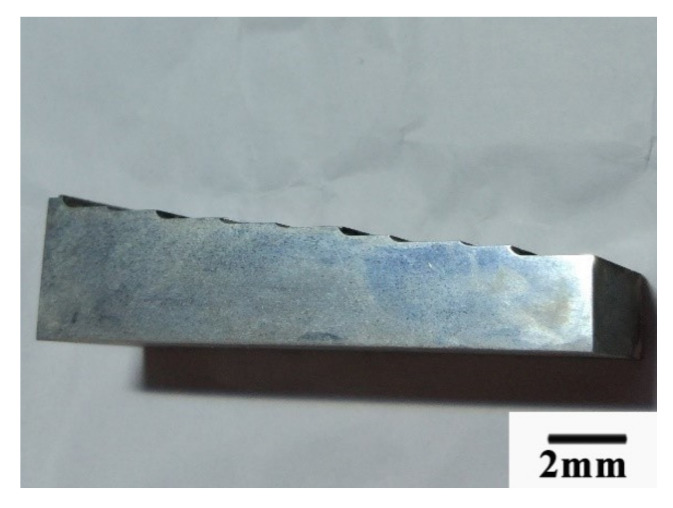
Material cut off from the nickel-based alloy.

**Figure 21:**
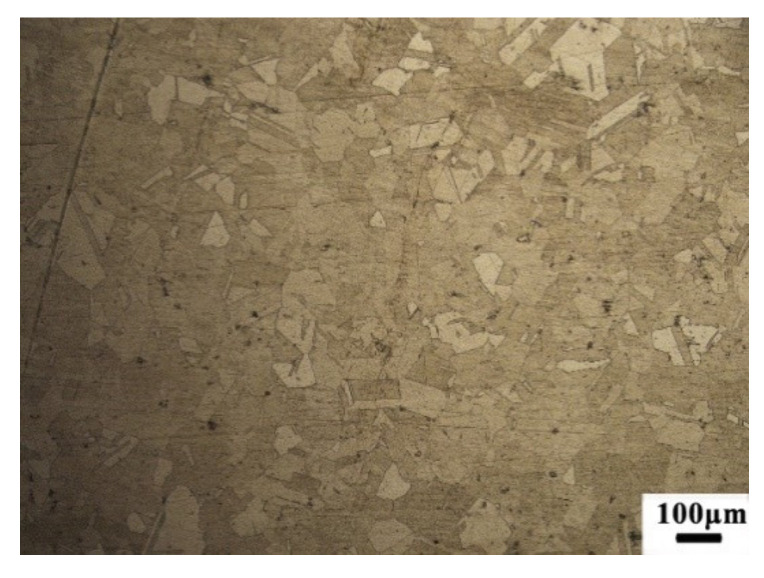
The buck material surface microstructure.

**Figure 22:**
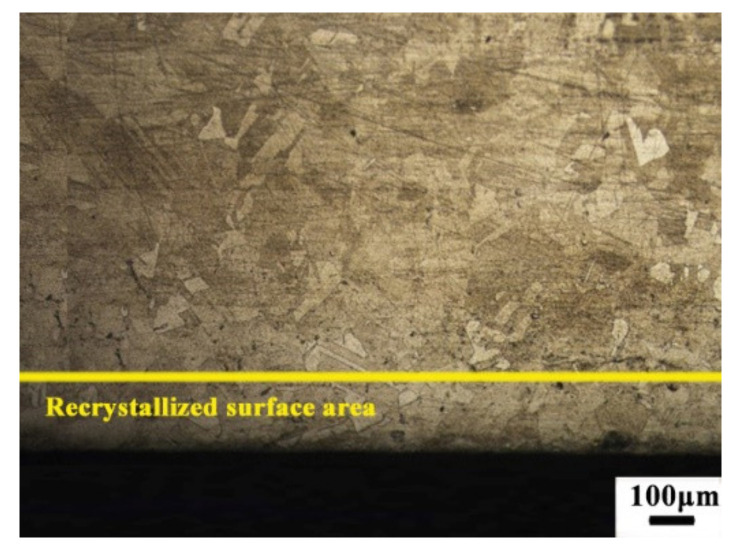
The side part microstructure of the machined surface.

After the dry milling process, the cut material was analyzed through a metallographic microscope. The dry milling process used nickel-based alloy and was cut off from the buck material, as shown in Figure 20. Figure 21 is the side part of the buck material microstructure. Figure 22 is the machined surface material microstructure. As Figure 22 shows, there was no surface burn on the workpiece material. The machined surface microstructure grain was smaller than the buck material, showing the machined surface was recrystallized after the dry milling process. The recrystallized surface was thin enough about 185 μm, which could be processed during the semi-finishing or finishing process.

**Figure 23:**
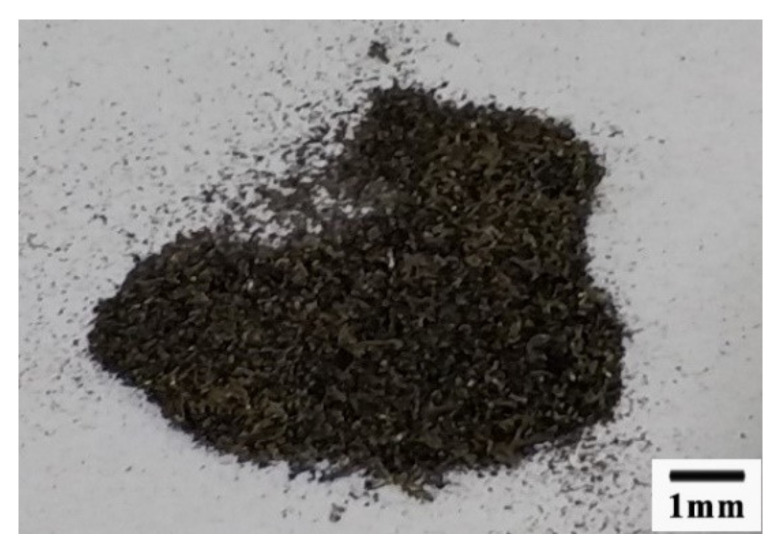
The chips formed when the cutting speed was above 600 m/min.

**Figure 24:**
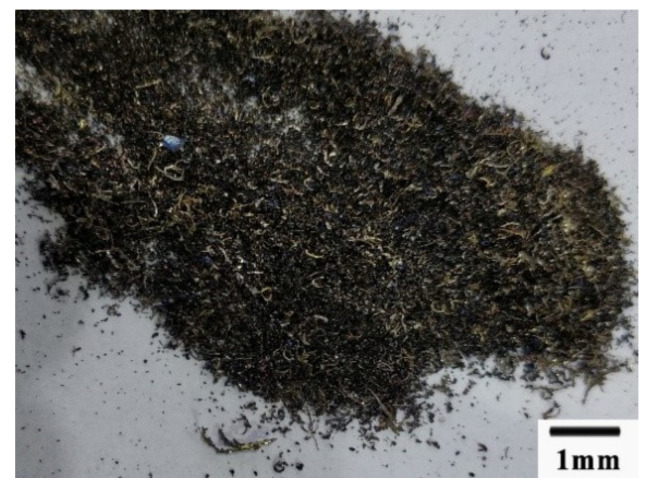
The chips formed when the cutting speed was below 600 m/min.

The chip’s form was different between the cutting speed higher than 600 m/min and cutting speed lower than 600 m/min. When the cutting speed was higher than 600 m/min, the chips looked like the power, as shown in Figure 23. When the cutting speed was lower than 600 m/min, the chips looked like a mixture of long comma chips and short comma chips, as shown in Figure 24.

## 4. Conclusions

In this research, the dry milling process of Inconel 718 was considered. A hypothesis about the heat generated in the cutting process made the uncut area materials easy to cut has been put forward, and several simulations and experiments about that process were undertaken. It can be concluded that with an increase in cutting speed, the cutting temperature increased at the same time. To generate enough heat, higher cutting speeds and higher heat-resistant milling tool materials should be put in to use. The ceramic material used could resist high temperatures and showed excellent performance during the machining process. The monolithic ceramic milling tool showed a higher material removal rate during the milling of difficult-to-cut materials.

Specific conclusions drawn are as follows.

(1) The ceramic material SiAlON could withstand the high temperature generated at the shear zone and the accumulated heat under high-speed machining conditions in the dry milling process.
(2) The heat generated in the high-speed milling process could cause a material softening effect on the nickel-based alloy. The average maximum temperature of the shear zone exceeded 1000 °C.
(3) The cutting force turning point was at 527.52 m/min; when the cutting speed exceeded that value, the cutting force began to decrease. When cutting speed continued to increase, the milling force continued to decrease. The milling speed increased the milling temperature to soften the workpiece material. Higher milling speeds could make the milling material removal rate to increase, further making the milling efficiency increase. From the metallographic microscope, the dry milling process had no surface burn on the processed nickel-based alloy surface.

## Figures and Tables

**Table 1:** The variation of feed per tooth.

Number	0	1	2	3	4	5	6	7	8	9
Feed per tooth (mm/tooth)	0.015	0.02	0.025	0.03	0.035	0.04	0.045	0.05	0.055	0.06

**Table 2:** The required cutting constants (unit is 1).

*K* *_tc_*	*K* *_te_*	*K* *_rc_*	*K* *_re_*	*K* *_fc_*	*K* *_fe_*
−538.26	−200.89	−5004.21	178.32	12973.39	274.29

**Table 3:** Milling simulation parameters and milling tool parameters.

Parameters	Value
Feed per tooth/*f_z_*	0.03 mm/tooth
Radial width of cut/*W_rd_*	5 mm
Initial temperature/*T_i_*	20 °C
Cutter diameter/*D*	12 mm
Core diameter/*D_c_*	9.02 mm
Number of flutes/*N*	6
Radial rake (tangential degrees*)/A_rt_*	−2°
Helical angle/*A_h_*	40°
Cutting-edge radius/*A_er_*	2
Radial primary relief angle/*A_rf_*	8°
Axial primary land/*A_pl_*	0.71 mm
Cutting speed/*V_C_*	300 m/min, 400 m/min, 500 m/min, 600 m/min, 700 m/min

**Table 4:** J–C (Johnson–Cook) failure parameters of Inconel 718.

Parameters	*d_1_*	*d_2_*	*d_3_*	*d_4_*	*d_5_*
Value	0.11	0.75	−1.45	0.04	0.89

**Table 5:** J–C parameters of Inconel 718.

Parameters	*A* (MPa)	*B* (MPa)	*C*	*n*	*m*	${\dot{\varepsilon }}_{0}\;$
Value	1241	622	0.0134	0.6522	1.3	1

**Table 6:** The mechanical property of Inconel 718.

Parameters	Density (g/cm^3^)	Melting Point (°C)	Thermal Conductivity (W/(m⋅°C))	Specific Heat Capacity (J/(kg⋅°C))	Poisson Rate
Value	8.24	1260–1360	14.7 (100 °C)	435	0.3

## Nomenclature

*a*	Cutting depth
*A*	Yield stress under reference deformation conditions (MPa)
*A_er_*	Cutting-edge radius
*A_h_*	Helical angle
*A_pl_*	Axial primary land
*A_rf_*	Radial primary relief angle
*A_rt_*	Radial rake angle (tangential degrees)
*B*	Strain hardening constant (MPa) *ε* is an equivalent plastic strain
*c*	Feed rate
*C*	Strain rate strengthening coefficient
*d_1_~d_5_*	Failure parameters
*D*	Cutter diameter
*D_c_*	Core diameter
*f*(*t*)	The function of interpolation curve
*f_z_*	Feed per tooth
*F_ave_*	Average milling force
*F_x_*	Force in the *x*-direction
*F_y_*	Force in the *y*-direction
*F_z_*	Force in the *z*-direction
${\bar{F}}_{q}$	Average cutting force in *x*, *y*, *z* directions (*q* = *x*, *y*, *z*)
${\bar{F}}_{qc}$	Cutting force coefficients in *x*, *y*, *z* directions (*q* = *x*, *y*, *z*)
${\bar{F}}_{qe}$	Edge force which does not depend on the chip thickness (*q* = *x*, *y*, *z*)
*h*	Chip thickness
*K_fc_*	Cutting force coefficients in the axial direction
*K_fe_*	Edge constants in axial direction
*K_rc_*	Cutting force coefficients in the radial direction
*K_re_*	Edge constants in radial direction
*K_tc_*	Cutting force coefficients in the tangential direction
*K_te_*	Edge constants in tangential direction
*m*	Thermal softening coefficient
*n*	Strain hardening coefficient
*N*	Number of flutes
*p*	Compressive stress
*q*	Mises stress
*S*	Spindle speed
*r*	Force reduction rate
*t*	The average temperature in the milling process
*T*	Current absolute temperature
*T_i_*	Initial temperature
*T_m_*	Melting temperature
*T_r_*	Reference temperature
*T**	Reference temperature
*V_c_*	Cutting speed
*W_rd_*	The radial width of cut
$\Delta {\bar{\varepsilon }}^{pl}$	The equivalent plastic strain of the increment step
$\dot{\varepsilon }$	Strain rate
${\dot{\varepsilon }}_{0}$	Reference strain rate
${\dot{\bar{\varepsilon }}}^{pl}$	Equivalent plastic strain
${\bar{\varepsilon }}_{f}{}^{pl}$	Strain failure value
*μ*	Friction coefficient
*τ*	Friction stress
*τ**	Maximum shearing stress
*σ*	Equivalent stress
*σ_B_*	Stefan-Boltzmann constant
*σ_n_*	Normal stress
*ω*	Fracture value
